# Reframing talent development in the intelligence age: reasoning from first principles

**DOI:** 10.3389/fpsyg.2026.1848779

**Published:** 2026-07-03

**Authors:** Ederick Stander, Ian Rothmann, Carla du Plessis, Sibusiso Mnxuma, Jordan Roothman, Joe Watson, Vesselin Popov

**Affiliations:** 1 Fitt Talent Solutions (FITT), Stellenbosch, South Africa; 2 Optentia Research Unit, North-West University, Vanderbijlpark, South Africa; 3 University of Cambridge Psychometrics Centre, Cambridge, United Kingdom

**Keywords:** artificial intelligence, Capability Approach, ecosystems, Information Processing Theory, organizational learning, talent development

## Abstract

This perspective paper examines how AI is reshaping talent management in the emerging intelligence age, where value creation depends on the ability to interpret, apply, and scale insight. Despite advances in talent analytics, learning, and skills models, current approaches are static and generally misaligned with the dynamic, AI-mediated future of work. We use three lenses–AI as an evolving, permeating, and performance-amplifying force–to investigate how this technology is transforming capability dynamics, decision-making, and performance distribution, while probing the limitations of existing talent practices. We put forward a framework–Assess, Access, Accelerate, and Amplify–that reframes talent management as an integrated approach to capability development.

## 1 Introduction

Talent management is in an age of converging disruption. Organizations are advancing on the mandate of talent datafication (Chamorro-Premuzic et al., 2017), investing in personalized, on-demand learning pathways (Liu and Yu, 2023), and transitioning toward skills-first workforce models (Jooss et al., 2024), in which talent mobility is central to the design logic. These developments do not occur independently; they reinforce one another, increasing both the pace of change and the complexity of coordination. This complexity is compounded by the integration of AI across HR value chains, which is reshaping how talent is assessed, developed, and deployed (Agnihotri et al., 2024).

Despite progress in areas such as talent analytics, learning, and skills development, operating modalities do not consistently translate into improved capability. In many cases, they remain only partially connected, limiting their overall effectiveness. What is currently failing in talent management systems is not the absence of sophisticated tools or frameworks, but the lack of coherence between them. Talent data is generated but not meaningfully translated into decisions; learning is personalized but weakly linked to evolving capability requirements; and skills architectures exist, yet rarely drive real talent mobility or deployment. As a result, organizations optimize in silos–assessment without activation, development without deployment, and insight without impact.

In what can be described as an emerging “intelligence age,” value creation depends not only on access to information but on the ability to interpret, apply, and scale insight effectively. Under these conditions, the challenge shifts from optimizing individual processes to ensuring they function as an integrated system (Faqihi and Miah, 2023). Organizations must continuously understand competence levels, connect individuals to opportunity, accelerate development, and translate this into performance at scale. AI sits at the center of this transformation, although its effects are neither uniform nor fully examined. Its application varies across organizational contexts, reshaping how capability is conceptualized, how decisions are made, and how work itself is structured. We interrogate this to unlock system value.

## 2 Theoretical grounding

The Capability Approach (CA) provides a human-centered and normative prism through which to examine capability development within organizations. It emphasizes individuals’ substantive agency to achieve valued forms of functioning, focusing on both what people are able to do and what they aspire to become (Nussbaum, 2011; Rothmann et al., 2025; Sen, 1999). Existing talent management literature has largely examined AI through operational, technological, or efficiency-oriented perspectives, with comparatively less attention given to how AI restructures capability development and human agency at a systemic level. This creates a theoretical gap in understanding how AI-enabled talent systems influence not only performance outcomes, but the conditions through which capability is deployed and amplified.

Secondly, Socio-Technical Systems (STS) Theory conceptualizes organizations as interconnected systems in which social and technical elements interact dynamically to shape work processes, organizational performance, and human outcomes. It is this co-existence which should be explored in depth; keeping in mind that, on balance, there are clear benefits for the expansion of AI into the advancement of human potential and talent (Zhang et al., 2025). Within people development, STS theory conceptualizes capability development as a systemic process emerging through the interaction between individuals, organizational structures, work design, and technological systems. As AI becomes increasingly embedded within these systems, it begins to reshape how capability is understood, how decisions are mediated, and how performance is distributed across organizations.

## 3 Three key lenses on AI and talent

AI will fundamentally transform the identification and development of talent, as long as there are clear tenets for how we think about AI, both as practitioners and researchers.

### 3.1 AI as an evolving force

Vrontis et al. (2023) argue that, whilst academic production in AI and intelligence systems has evolved significantly, our understanding of the integrated power of these technologies across ecosystems (at the individual and organizational layer) remains limited. AI introduces a dynamic and continuously evolving capability environment, in contrast to the relatively stable contexts assumed in traditional talent management models. AI systems improve through data exposure, iterative learning, and ongoing model refinement, resulting in capabilities that shift over time rather than remaining fixed.

From a talent management and development perspective, this redefines the nature of skills and capability. Rather than being treated as stable attributes developed through periodic interventions, skills become fluid, context-dependent, and closely linked to human-AI interaction. In this environment, the Dynamic Capabilities Framework offers an anchor point, referencing organizations’ “ability to integrate, build, and reconfigure internal and external competences to address rapidly changing environments” (Teece et al., 1997, p.516). Contemporary research in Human Resource Management and Organizational Psychology also points to this importance of adaptability, continuous learning, and augmented decision-making as core components of effective performance in AI-enabled environments.

Competency frameworks, role architectures, and learning systems are typically designed around relatively stable definitions of work and capability, updated at discrete intervals. In rapidly evolving AI contexts, such approaches risk becoming misaligned with actual capability requirements, creating a lag between development efforts and performance demands. This will demand a much more focused intentionality on the part of the practitioner, to whom context-specific configuring at the very early stages of design will be important (Jia et al., 2018).

### 3.2 AI as a permeating force

Artificial intelligence is increasingly permeating decision-making across talent management processes, shaping how decisions are informed, made, and executed (Hunkenschroer and Luetge, 2022; Jacob Fernandes França et al., 2023). From talent acquisition and performance management to workforce planning and internal mobility, AI is becoming embedded in the flow of work, influencing choices through data-driven insights and recommendations.

This changes how decisions are made in practice. Individuals are no longer operating as isolated decision-makers but are working in environments where judgment is informed and shaped by AI-enabled inputs. Effective performance therefore depends not only on domain expertise, but on the ability to interpret, apply, and critically evaluate these inputs in context. In organizations, it suggests an increasingly sophisticated exchange between human and machine, evolving STS Theory and how we make sense of organizational development.

This shift places new demands on talent practices, challenging traditional approaches that have largely been designed around individual competence and accountability, with limited consideration for how technologically embedded environments shape decision-making and performance. Learning interventions must extend beyond the development of discrete skills toward cultivating the judgment and adaptability required to operate effectively in increasingly data-informed contexts. Consequently, organizations must establish appropriate policy infrastructures and governance guardrails, while simultaneously upskilling employees and continuously adapting to rapid technological developments (Van Zyl, 2026a). In this context, talent management must broaden its focus from developing individual expertise alone to enabling stronger judgment, informed decision-making, and effective interaction with AI-enabled systems and processes.

### 3.3 AI as a force multiplier of performance

Artificial intelligence is not only changing how work is performed, but also how performance is generated and distributed. By augmenting cognitive tasks, accelerating execution, and enabling access to insight at scale, AI acts as a force multiplier–amplifying the output of individuals and teams.

For talent management, this creates a shift in how performance should be understood and developed. Traditional approaches often assume relatively linear relationships between effort, capability, and output. In AI-enabled environments, performance becomes more variable and less predictable, shaped by how effectively individuals can work with and through intelligent tools. What this requires, naturally, are far smarter, opinionated systems which can draw from inputs, turning fragmented information into integrated insight (Asfahani, 2024; Martorell et al., 2025). Standardized learning pathways, uniform competency expectations, and broad-based development interventions may be insufficient in environments where performance is increasingly differentiated. Instead, there is a need for more targeted approaches that identify where amplification is occurring, support individuals in leveraging it effectively, and address emerging gaps in a proactive way.

Taken together, these lenses point to a common conclusion: the assumptions underpinning talent management are increasingly misaligned with the realities of the intelligence age. Capability is no longer stable, decision-making is no longer solely human, and performance is no longer evenly distributed. Yet many existing approaches remain anchored in static frameworks, linear development pathways, and fragmented practices that struggle to operate coherently under these conditions. In accordance with STS Theory, the risk for disharmony is the disconnect between the pace of evolution of AI, and the capability of people to adopt and scale these systems in their ecology (Ang et al., 2025).

Addressing this gap requires more than incremental improvement–it requires rethinking talent management from first principles. Rather than adapting existing models, the focus shifts to a more fundamental question: How is capability continuously understood, accessed, developed, and translated into performance in dynamic, AI-enabled environments? The model that follows is grounded in this logic, framing talent management around four interdependent drivers: Assess, Access, Accelerate, and Amplify.

## 4 4 A model: a first principles framework

First-principles reasoning is a method of thinking that decomposes complex problems into their foundational elements and reconstructs solutions from basic truths, rather than relying on analogy, convention, or inherited practice. Widely associated with entrepreneurship and engineering (Tovstiga, 2023), first-principles thinking also offers value within talent management by challenging traditional assumptions about capability, performance, learning, and organizational design. From this perspective:

- Talent systems should be designed around capability and adaptability, rather than static roles or hierarchies.
- Performance emerges through the interaction between people, systems, and technology, not individual competence alone.
- AI creates value only when human judgment and technological capability are effectively integrated.
- Learning should cultivate adaptability and decision-making, rather than merely developing discrete skills.
- Integrated talent ecosystems produce stronger capability outcomes than fragmented talent practices.

This shifts the architecture of talent development itself: from a largely linear and programmatic process to a more integrated, dynamic, and system-level capability. Theoretically grounded in the Capability Approach (CA), Socio-Technical Systems (STS) Theory, and the Dynamic Capabilities Framework, this paper poses four baseline probes for talent development in the intelligence age:

- To what extent can capability be leveraged without true understanding?
- What value does capability hold if it cannot be reliably accessed?
- How sustainable is performance when capability remains static?
- Can performance endure in environments that do not actively enable it?

Furthermore, this paper introduces the 4A Model of Capability Velocity as an original conceptual framework structured around the interconnected principles of Assess, Access, Accelerate, and Amplify, intended to guide a more coherent and capability-driven approach to talent management.

### 4.1 Assess

Talent assessment must be intentional: identifying the right capability signals, at the right time, for the right strategic domains (Yildiz and Esmer, 2023; Jooss et al., 2024). In the intelligence age, assessment must move beyond narrow, once-off, and role-bound assessments toward richer and more responsive approaches that focus on capabilities that matter in the current environment. This includes agility (World Economic Forum, 2025), continuous learning (Beier et al., 2025; World Economic Forum, 2025), adaptability, and judgment (Hossain et al., 2025). The aim is not simply to assess faster or at greater volume, but to assess more meaningfully. In this sense, assessment should function as a genuine development diagnostic partner to the organization whilst at the same time offering meaningful insights and growth pathways to the individual being assessed (Stander et al., 2022; Tusquellas et al., 2024).

Assessments must be designed for agility–capturing transferable, fit-for-purpose competencies with both reliability and true construct validity. Done well, they produce talent-to-value heatmaps and evolve from static events into continuous systems of predictive and strategic insight (Sparkman, 2025).

**FIGURE 1:**
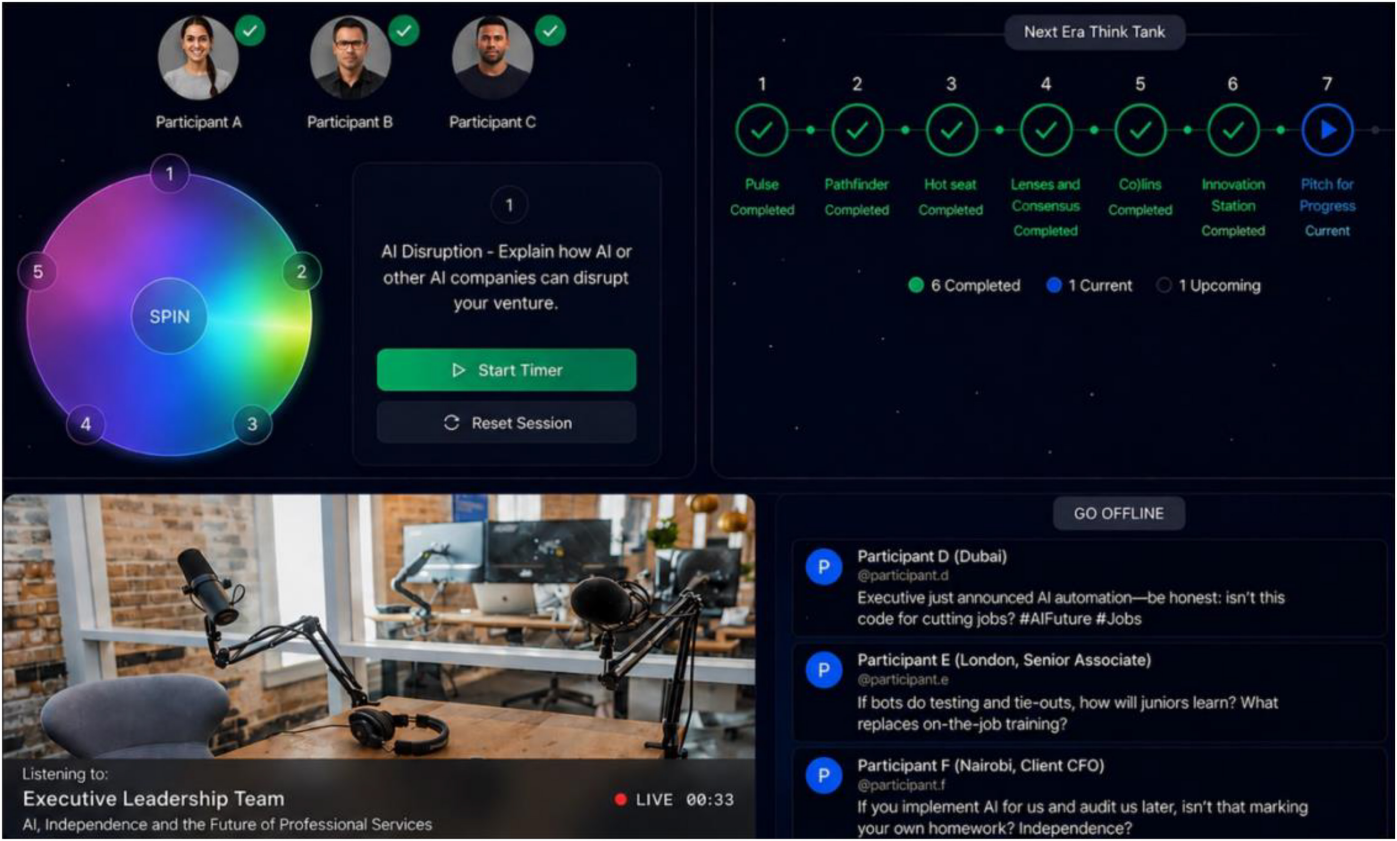
Example extract from the FITT^®^ multi-method immerse simulation assessment platform for model component “Assess.”

Figure 1 illustrates how our work applies the Mulitrait-Multimethod (MTMM) approach, integrating multiple assessment methods to develop a multidimensional understanding of talent and capability. This approach, first conceptualized in the pioneering work of Campbell and Fiske (1959), enables richer capability inference while reinforcing both convergent and discriminant validity across assessment outcomes. More simply, we use different assessment protocols, configured for sector, level, application context and function, to build a coherent view of an individual’s key strengths, development gaps, preferences and aspirations. This serves as the foundation for a granular and deeply personalized lens on growth opportunities. Digital tools and advancing agentic AI frameworks offer high utility in presenting assessments in journeyed format to candidates, supporting an engaging and learning-enriched experience.

### 4.2 Access

Capability insights can only be translated into talent mobility when accessed through a coherent infrastructure. Many organizations still operate with fragmented talent data spread across systems, teams, and processes (Asfahani, 2024; Shan and Wang, 2024). These fragmented data environments prevent organizations from connecting assessment information and other potentially valuable insight to strategic decisions around succession, workforce planning, internal mobility, risk signals, and development needs (Madhumithaa et al., 2025). The challenge, then, is to move from fragmented data to integrated talent intelligence. This is where talent analytics platforms, dashboards, and connected systems become important.

However, the solution is not platform accumulation or even data centralization per se. HR analytics literature shows that data only becomes strategic when it is institutionalized in a way that makes it possible to connect information across the talent ecology and support richer dialogue and better decision making (Touriano et al., 2023). Enterprise-grade infrastructure and talent dashboards are not inherently transformative–data and digital platforms should be seen as enabling conditions rather than ends in themselves. It is not just about having data available; it is about making talent insight usable, shared, and strategically meaningful (Faqihi and Miah, 2023).

Organizations need tools that are secure, flexible, always-on, and capable not just of displaying information, but of enabling sense-making. Access alone, however, is not insight. Without interpretation, governance, and decision capability, integrated systems risk entrenching fragmentation rather than resolving it. This demands a design pivot–from systems of record to increasingly intelligent, opinionated architectures that actively guide decision-making in dynamic contexts (Jooss et al., 2024; Asif et al., 2025).

From the vantage point of Information Processing Theory (Galbraith, 1974; Tushman and Nadler, 1978), Figure 2 illustrates how organizational effectiveness depends increasingly on the ability to acquire, integrate, and translate talent intelligence into informed capability decisions. This remains compatible with the Capability Approach, which emphasizes human agency and the conversion of resources into valued outcomes, as well as Socio-Technical Systems Theory, which views capability and performance as emerging through the interaction between people, systems, and technology. We have developed the Talent IQ system, an integrated Talent Management as a Service (TMaaS) platform designed to support real-time talent intelligence, mobility, succession planning, and workforce decision-making through connected and continuously updated capability data.

**FIGURE 2:**
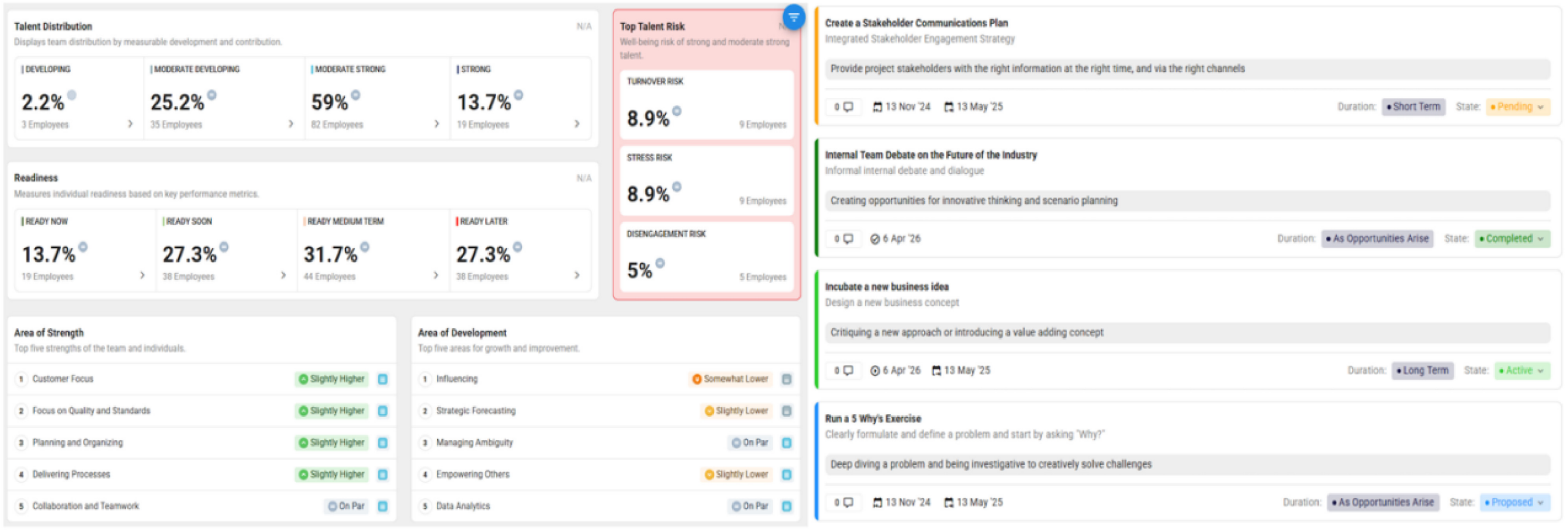
Example extract from a FITT^®^ Talent IQ deployment as part of model component “Access.”

### 4.3 Accelerate

Accelerate is the activation component–converting insight into capability through curated, strategically aligned learning pathways. Assess defines the need; Access connects the intelligence; and Accelerate delivers targeted development. This is where AI may be most productively understood as an enabling layer for next-generation talent development: it can support tailored pathways (Tusquellas et al., 2024; Beier et al., 2025), adaptive feedback, and more continuous developmental support (Tusquellas et al., 2024). Current market solutions strive to provide development systems that make learning more personalized and scalable (Parasa, 2025). However, a targeted approach is not the primary argument of Acceleration; what matters most is whether these interventions build capabilities that are genuinely tied to strategy and whether they help individuals grow in ways that endure beyond the immediate intervention (Madhumithaa et al., 2025).

Recent work on personalized HRM is useful here because it suggests that data-informed differentiation may represent a next generation of HR practice, but only if it remains tied to fairness, transparency, and meaningful developmental outcomes rather than hyper-individualized optimization. Development should therefore be signature, metric-informed, and responsive, but not reduced to algorithmic recommendation. Otherwise, acceleration risks becoming another form of optimization without depth. In the realm of AI, Van Zyl (2026b, p. 7) offers a sober warning, with guidance: “Capability develops through active engagement, and not through passive consumption of content. Psychological agency requires doing the work, not having it done for you. This creates the central paradox of AI assistance: systems designed to help may systematically undermine the capacities enabling flourishing. As such, there is a need for a framework that identifies which specific human capacities are at risk under algorithmic conditions, how these capacities develop and how AI system design either supports or systematically erodes them.” Technology is expanding what is possible in learning through tailored guidance, bespoke development, and highly curated content. The response, however, must be human-led: an integrated design that keeps the development of the individual front and center.

**FIGURE 3:**
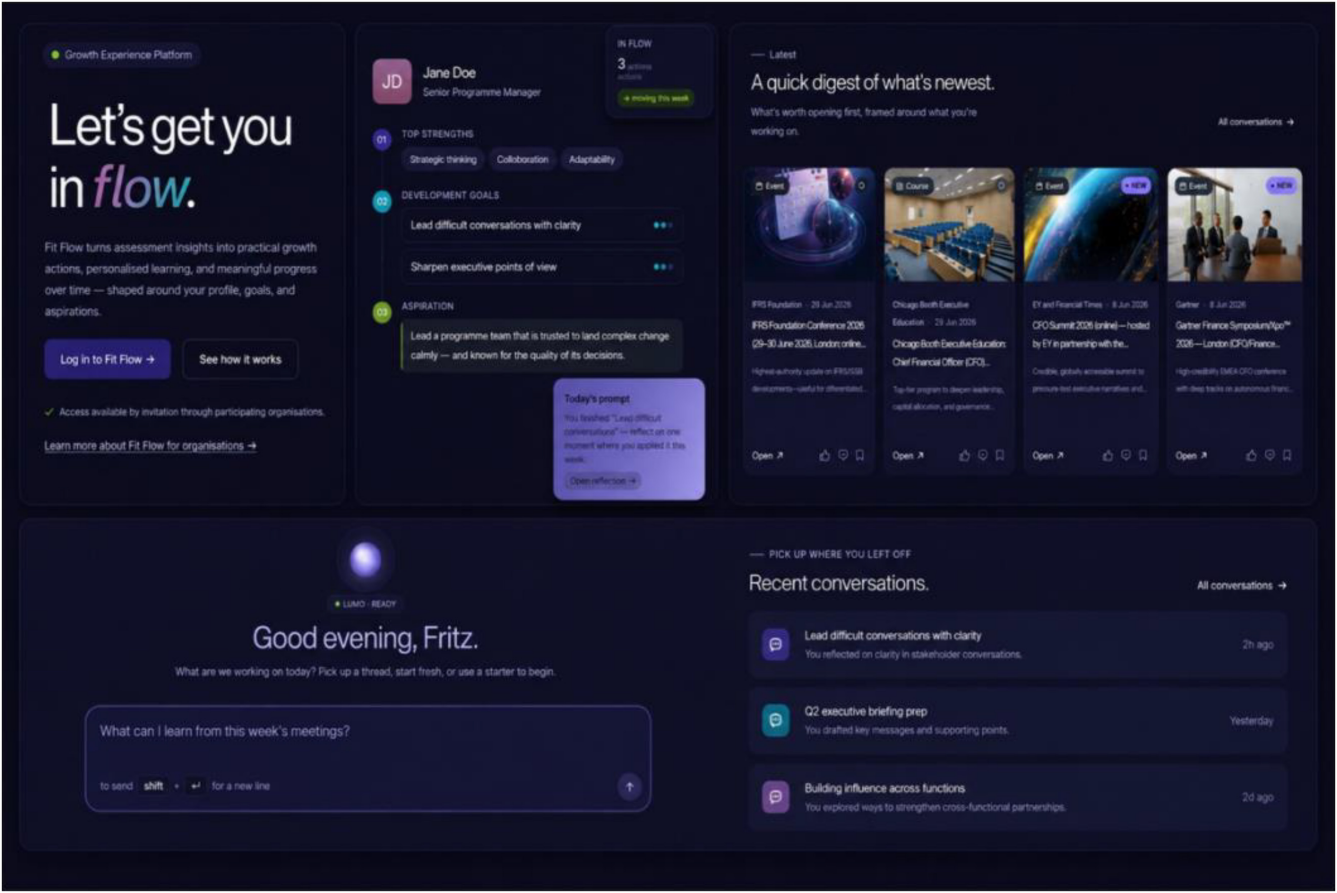
Example extract from the FITT^®^ FLOW as part of model component “Accelerate.”

As shown in Figure 3, our practice supports capability acceleration through FLOW, our proprietary Growth Experience Platform (GXP), which is designed to enable capability development within the flow of work through personalized learning, applied performance, and continuous developmental progression. The platform shifts learning beyond passive content consumption toward the active application of knowledge, supporting more rapid skill acquisition, improved decision-making, and capability development embedded within real work contexts. Drawing from the model of Experiential Learning Theory by Kolb (1984), this approach conceptualizes learning as an iterative process through which knowledge is developed through experience, reflection, application, and experimentation. In this way, capability acceleration becomes closely linked to continuous practice and real-world application, rather than the passive acquisition of information alone.

### 4.4 Amplify

Amplify ensures talent sustainability through end-to-end systems that compound value over time. It is where the model becomes fully systemic–and where most organizations fall short. Tools and data deliver value only through the people and processes that apply them; amplification is therefore inherently process driven. It is concerned with ensuring that talent insights are intuitive, embedded in business strategy, owned by leaders, and translated into repeatable cycles of assessment, development, deployment, and review (Douglas et al., 2022; Rožman et al., 2022; Bandura, 2023). It also requires investment in the capability of those responsible for driving the system forward, including HR professionals, business leaders, and communities of practice that can sustain learning and adaptation (Dima et al., 2024).

In this sense, amplification is what prevents talent management from remaining static. It turns isolated interventions into self-reinforcing processes that can respond to changing organizational demands and shifts. This is especially important in AI-enabled environments, where the pace of change can make static talent models obsolete very quickly (Rožman et al., 2022). Amplify requires an ecosystem view, since AI-enabled talent systems increasingly depend on external platforms, vendors, and networked forms of expertise. Morelli (2019) encourages critical evaluation of particularly external partners and products, arguing the importance of understanding the AI tools brought into business ecosystems. In this respect, amplification is not simple scale; it is the institutionalization of responsible human–AI capability. Without that integrative layer, organizations risk replacing fragmented talent practices with fragmented technologies (Touriano et al., 2023; Jooss et al., 2024; Van Zyl, 2026b).

**FIGURE 4:**
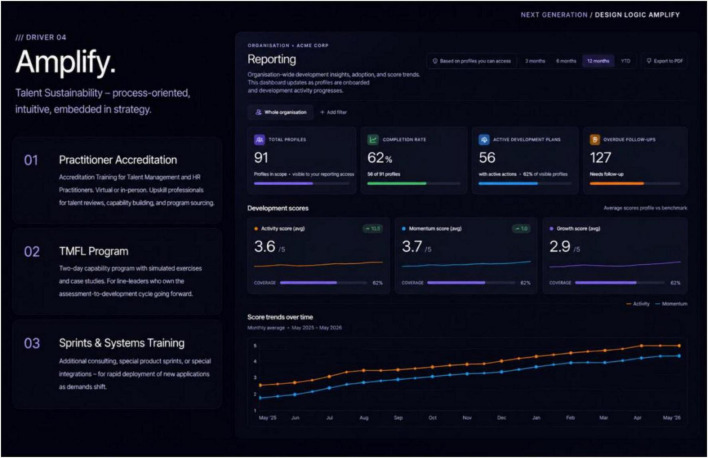
Example extract from an actual organizational use case as part of model component “Amplify.”

As shown in Figure 4, amplification becomes possible when capability approaches are sustainable, process-oriented, and embedded within the strategic and operational fabric of the organization. In practice, this requires reinforcing continuity through established operating rhythms and organizational cadence. To support this, organizations may deploy a range of continuity interventions, including practitioner training, line manager enablement, system integration activities, and user-focused capability workshops designed to sustain adoption, alignment, and long-term capability progression. This perspective aligns with Organizational Learning Theory (Argyris and Schön, 1978), which proposes that organizations develop sustainable capability through the continuous creation, integration, sharing, and application of knowledge across systems, routines, and practices.

## 5 Conclusion and future research

The intelligence age is reshaping the foundations of talent management. As AI becomes increasingly embedded across organizational systems, traditional approaches built around static competency models, fragmented development practices, and episodic interventions appear increasingly insufficient for the complexity and dynamism of contemporary work. Capability is becoming more fluid, decision-making more technologically mediated, and performance more unevenly distributed across individuals and systems.

Drawing on the Capability Approach, Socio-Technical Systems Theory, Information Processing Theory, Dynamic Capabilities Theory, and Organizational Learning Theory, this paper proposed the 4A Model of Capability Velocity as an integrated framework organized around Assess, Access, Accelerate, and Amplify. Collectively, these dimensions reposition talent management from fragmented HR practices toward a more adaptive, intelligence-enabled capability system.

Future research should empirically examine the applicability of the model across industries and organizational contexts, while also exploring the long-term implications of AI-enabled talent systems for capability development, workforce adaptability, governance, fairness, and human agency. Subsequent studies could also develop measurable indicators for each dimension of the model and validate them using employee surveys, longitudinal field studies, AI system data, or experimental designs. Ultimately, sustainable performance in the intelligence age may depend less on isolated talent interventions and more on the organization’s ability to integrate human capability, organizational learning, and technological intelligence into coherent and continuously evolving systems.

## Data Availability

The original contributions presented in this study are included in this article/supplementary material, further inquiries can be directed to the corresponding author.
